# Characterisation and Quantification of Upper Body Surface Motions for Tidal Volume Determination in Lung-Healthy Individuals

**DOI:** 10.3390/s23031278

**Published:** 2023-01-22

**Authors:** Bernhard Laufer, Fabian Hoeflinger, Paul D. Docherty, Nour Aldeen Jalal, Sabine Krueger-Ziolek, Stefan J. Rupitsch, Leonhard Reindl, Knut Moeller

**Affiliations:** 1 Institute of Technical Medicine (ITeM), Furtwangen University, 78054 Villingen-Schwenningen, Germany; 2 Department of Microsystems Engineering, University of Freiburg, 79085 Freiburg, Germany; 3 Department of Mechanical Engineering, University of Canterbury, Christchurch 8041, New Zealand; 4 Innovation Center Computer Assisted Surgery (ICCAS), University of Leipzig, 04109 Leipzig, Germany

**Keywords:** wearables, smart clothing, respiratory parameters, inertial measurement units, strain gauges, movements upper body, tidal volume

## Abstract

Measurement of accurate tidal volumes based on respiration-induced surface movements of the upper body would be valuable in clinical and sports monitoring applications, but most current methods lack the precision, ease of use, or cost effectiveness required for wide-scale uptake. In this paper, the theoretical ability of different sensors, such as inertial measurement units, strain gauges, or circumference measurement devices to determine tidal volumes were investigated, scrutinised and evaluated. Sixteen subjects performed different breathing patterns of different tidal volumes, while using a motion capture system to record surface motions and a spirometer as a reference to obtain tidal volumes. Subsequently, the motion-capture data were used to determine upper-body circumferences, tilt angles, distance changes, movements and accelerations—such data could potentially be measured using optical encoders, inertial measurement units, or strain gauges. From these parameters, the measurement range and correlation with the volume signal of the spirometer were determined. The highest correlations were found between the spirometer volume and upper body circumferences; surface deflection was also well correlated, while accelerations carried minor respiratory information. The ranges of thorax motion parameters measurable with common sensors and the values and correlations to respiratory volume are presented. This article thus provides a novel tool for sensor selection for a smart shirt analysis of respiration.

## 1. Introduction

The measurement of tidal volumes based on respiration-induced surface motions of the upper body has been a part of research for decades. Pioneers in this field of research were Konno and Mead in the 1960s [1]. Although the motivation was different at that time, the potential and benefits combined with surface motion measurements were already apparent, and thus, many other research approaches and studies followed over the years. Unfortunately, the success of most studies has been marginal; only two measurement techniques have been able to establish themselves in clinical practice or homecare and are used sporadically in present days. These two techniques are the optoelectronic plethysmography (OEP) [2,3] and the respiratory inductance plethysmography RIP [4]. The OEP is based on an optical motion tracking system (MoCap) that measures respiration induced movements on the upper body to determine respiratory parameters, while the RIP measures changes in cross sections at the upper body inductively to obtain respiratory parameters such as the tidal volume. However, both of these methods are associated with disadvantages. The OEP involves a complex, non-wearable system and high costs, while the RIP struggles with inaccuracy of the measurement results [5]. Therefore, despite all previous efforts, tidal volume measurement is still based on airflow measurements via spirometry [6,7] or body plethysmography [8,9]. In other words, the determination of respiratory parameters via surface motions of the upper body is still a part of research.

Whereas in the past the motivation was rather the inaccuracy of other existing measurement methods (including spirometry), today the focus is more on a measurement comfortable and convenient for persons being examined. However, even if the motivation changed, the goal is still the same: avoiding flow measurements. Flow measurements generally require the use of a face mask, or the person examined must breathe through a mouthpiece while the nose is blocked by a nose clip. While present flow sensors can measure laminar airflow accurately, facemasks or mouthpieces can be uncomfortable, especially in long term measurements, and may affect the measurement results itself [10,11]. Thus, a measurement method as an alternative to flow measurement would be beneficial then as now.

The existence of a relationship between breathing and surface movements of the upper body has always been obvious. The fundamentals and physiological basis of respiration induced movements of the human upper body were already analysed and published as early as 1848 by Sibson et al. [12], using simple techniques available at that time. Later, Wade et al. performed a more sophisticated analysis of the movements in 1954 [13]. Since then, new and improved measurement techniques, especially optical measurement techniques (MoCap or laser scanners) or other imaging techniques (magnetic resonance imaging or computer tomography), have provided a much better understanding of respiration induced movements; however, the fundamentals provided by Wade et al. are still valid.

In last decades, new sensors and sensor technologies, such as miniaturized sensors, have opened up many new opportunities and applications [14,15]. The improved implementation of miniaturized sensors in garments and their enhanced accuracy have enabled a variety of new applications. Smart shirts and intelligent garments are on the rise and are increasingly being used in medical diagnostics and therapy control as well [16,17,18,19]. While mobile solutions in cardiovascular monitoring, e.g., heart rate monitoring, have been successfully used for some time, respiratory monitoring systems are mostly limited to monitoring respiratory rate [20,21,22,23,24,25,26], which can be measured with sufficient accuracy for clinical purposes.

However, measurement methods in respiratory diagnostics with smart shirts are not very advanced in accurately determining respiratory volume, and a real breakthrough has not yet been achieved. Some studies focused on inertial measurement units IMUs [22,26,27,28] that can measure accelerations and tilt angles, others on strain gauges [29], or on optical encoder systems, such as CiMeD belts [30] that measure circumferential changes on the upper body by optical encoders.

A smart shirt for monitoring vital parameters is the Hexoskin Shirt (Montreal, QC, Canada) [31]. Various studies evaluated this smart shirt [32,33,34], and the results of heart rate and respiratory rate measurements are in a clinically relevant range. However, when measuring respiratory volumes (minute volumes), errors ranging from 9.5% to 19.6% and even up to 41% during exercise were found compared to the reference devices used, which is outside the clinically relevant range. In summary, it is apparent that some of the new developments are promising but still subject to inaccuracies.

The development of a smart shirt approach for respiratory analysis requires the range of motion and information content of various locations on the thorax to be precisely understood. To analyse the theoretical possibilities of tidal volume measurement via surface motions of the upper body, this research analysed in detail the movements of the upper body, captured by a motion tracking system. Based on the movements of the motion tracking markers, the range and information content of various motion parameters (such as upper body circumference changes, local extensions, tilt angles and accelerations) were captured at the positions of the MoCap markers and were tested and evaluated for theoretical suitability to determine tidal volume. Thus, this study provides a support for sensor selection, representing the changes of the parameters to be measured on the upper body and offering the respective correlation to the respiratory volume. A specific selection of a sensor-set was not carried out, as this process is individually dependent on the types of sensors desired.

## 2. Materials and Methods

### 2.1. Sensors Targeted by the Study

Optical tracking or laser systems directly provide the spatial data of the object under examination, which in the given situation is the human upper body. Since these systems are usually associated with higher costs and can hardly be integrated into a smart shirt, other sensors must be employed to detect surface movements of thorax and abdomen, such as inertial measurement units (IMUs), strain gauges, or optical encoders in CiMeD-belts. This work focuses on analysing the range of changes in respiration-induced motion parameters: spatial positions, accelerations, distance variations, circumferential changes at the upper body and changes in tilt angles, which can be captured with the mentioned sensors. The results of this work provide the basis for any other sensor technology used to determine one of these motion parameters. 

An IMU with 9 degrees of freedom (DOF) combines a 3-axis gyroscope, a 3-axis accelerometer and a 3-axis magnetometer. The gyroscope measures angular rates in the x-, y- and z-direction, the accelerometer accelerations in x-, y- and z-direction, and the magnetometer the magnetic field or magnetic dipole moment in all 3 directions. Thus, for the given area of application, tilt angles and acceleration data can be predominantly used from IMUs, integrated into a smart shirt, e.g. [18]. A survey of applications and methods on IMUs placed on the upper body to monitor respiration can be found in Rahmani et al. [27]. 

Looking at strain gauges, there are many strain gauges with different properties on the market, some of which are specially tailored to the particular application. In order to determine strains of textiles by means of strain gauges, in contrast to strain gauges for other applications (carrier films of the strain gauges are made of acrylic resin, epoxy resin, phenolic resin or polyamide), larger strain ranges must be expected so that the strain gauge can expand with the textile. It would be ideal if the textile carrier material of the strain gauge had similar elongation properties to the textile under investigation, the smart shirt itself. Larger strain ranges are usually found in textile strain gauges, whose sensor wires are woven directly into the textile. These strain gauges could be used in smart shirts to obtain changes in distance, such as distances between MoCap markers. 

Putting the attention on circumferential changes, which are basically as well a distance change or expansion, then, due to the dynamic character and circular arrangement and the large dimension of these changes (changes up to 100 mm and more), their determination requires a special measuring principle. These dynamic circumferential changes of the upper body can be determined inductively, for example in RIP [35], by textile strain gauges [36], or by optical encoders via CiMeD belts [30]. 

Caution must be taken to ensure that the measurement system only slightly inhibits the expansion of the upper body during respiration to allow examinations of patients with pulmonary disease. If the restoring forces are too high, the compliance of the upper body will increase, and the measurement results themselves will be falsified. However, none of the mentioned sensors would interfere significantly with the measurement itself, and all would allow comfortable measurement of respiratory movements. 

### 2.2. Measurement Setup

**Figure 1:**
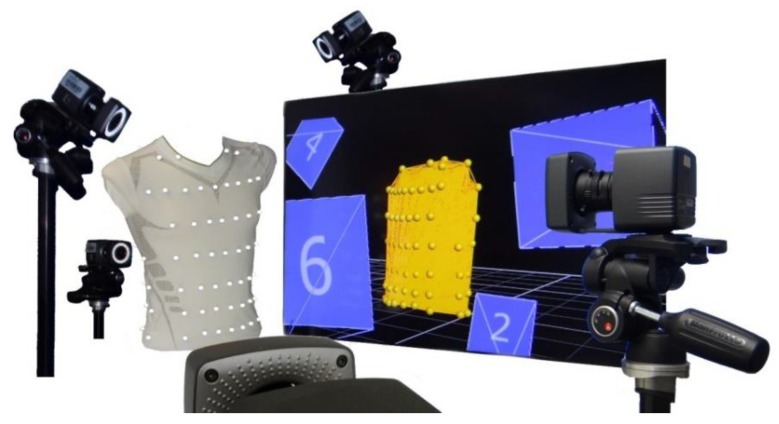
Schematic sketch of the MoCap system.

**Figure 2:**
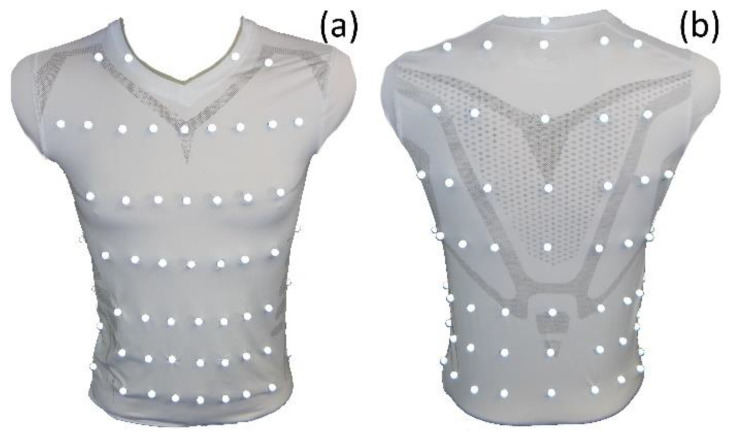
Compression shirt with 102 reflective MoCap markers—ventral view (**a**) and dorsal view (right). The reference point in dorsal view (**b**) is the uppermost MoCap marker on the collar of the shirt.

To analyse respiration-induced movements of the upper body accurately, a MoCap (Bonita, VICON, Denver, CO, USA) with nine infrared cameras (VICON Bonita B10, Firmware Version 404) was utilised. A schematic sketch of the MoCap system is illustrated in Figure 1. The participating subjects wore a tight compression shirt with a total of 102 reflective motion capture markers attached (Figure 2). Forty-eight markers were fixed ventral on the shirt, 18 lateral, and 36 dorsal—in 8 different heights. The uppermost MoCap marker (Figure 2b) on the collar of the shirt is located at the level of the cervical vertebrae C6 and serves as a reference point, since this location at the cervical spine is barely exposed to any respiratory movement. Thus, during further measurements, movements that are not respiration-induced can be corrected by this reference point.

Height 2 was in the level of the thoracic vertebra T1, and respectively in height of the clavicular. Height 3 was at the level of T4, while height 4 was at the level of T7, caudal below the scapula. Height 5 was at the level of thoracic vertebra T11, and height 6 was at the level of lumbar vertebra L1, just at the caudal end of the arcus costalis. Height 7 was at the level of L3, and finally, height 8 was at the level of L5. However, these descriptions of heights are only approximations that varied depending on the body shape, and especially the body height, of the participants wearing the compression shirt.

The subjects performed tidal volume measurements via a spirometer (SpiroScout and LFX Software 1.8, Ganshorn Medizin Electronic GmbH, Niederlauer, Germany) while they wore the compression shirt and were surrounded by the MoCap cameras. Both measurements were performed simultaneously. The spirometer data were utilised as reference values for tidal volumes. Flow and volume data were obtained by the SpiroScout with a sampling frequency of 200 Hz. The sampling frequency of the MoCap system was set to 40 Hz. The VICON Nexus Software (Version 1.8.5.6 1009h, Vicon Motion Systems Ltd., Denver, CO, USA) was used to process the raw data and to estimate the location of the markers that were optically obscured and finally, the spatial positions of all markers at each point in time were transferred to MATLAB (R2021a, The MathWorks, Natick, MA, USA), for subsequent analysis.

**Figure 3:**
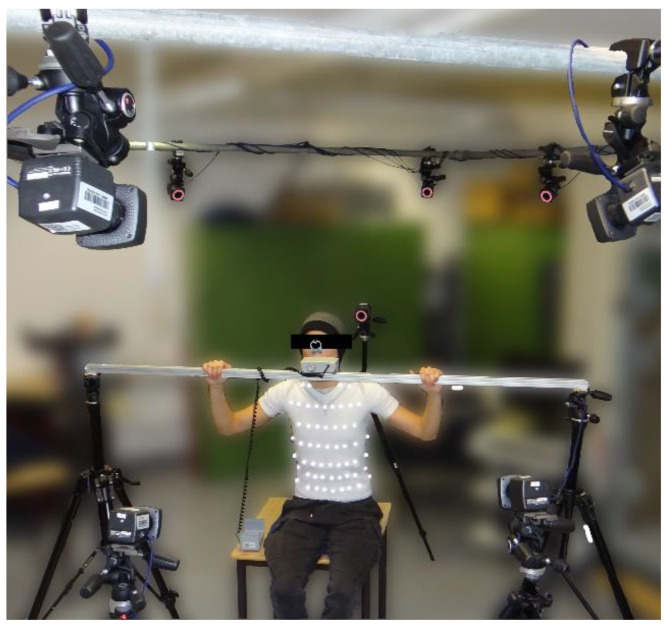
Measurement setup: A subject wearing the compression shirt with MoCap markers, breathing through the spirometer which was fixed on the rigid mount and surrounded by the MoCap cameras.

The data were obtained from subjects in a sitting position (Figure 3). To reduce movements of the upper body, which are not related to respiratory activity such as bending or twisting, the spirometer was fixed on a rigid hold to the height of the subject’s mouth when sitting upright. Thus, the subjects performing the measurement did not move their head and upper body significantly, and the obtained movement data were nearly limited to respiration-induced movements.

### 2.3. Participants and Respiratory Manoeuvres

All measurements were done in accordance with the tenets of the Helsinki Declaration. The ethical approval for this low-risk study was obtained from the Human Ethics Committee of the University of Canterbury HEC 2019/01/LR-PS and the Ethikkommission of the Furtwangen University. Before the measurement, the subjects received a full explanation of the study and were informed about any minor risks, even when only low risks were associated with these measurements and very unlikely to happen. A written informed consent was collected from each subject. The subjects were assured that they can remove the mouthpiece of the spirometer at any time and stop without penalty if even the slightest discomfort occurred. 

Sixteen lung healthy subjects voluntarily participated in the measurements—three women and thirteen men. The subjects’ average age, weight and height were 25.7 ± 2.2 years, 69.4 ± 2.0 kg and 1.76 ± 0.02 m, respectively. For more details on the subjects, please refer to Table 1. Additionally, the vital capacity, which is defined as the maximal volume of air the subject can inhale after total exhalation, was measured with the SpiroScout spirometer by means of a Tiffeneau test [37]. A Tiffeneau test is a pulmonary function test, typically used to determine volume that can be maximally exhaled within one second after a maximum inspiration (termed forced expiratory volume per second). During a Tiffeneau test, subjects exhale maximally, and then they inhale maximally, before subsequently exhaling totally as quickly as possible. To enhIn addition to the forced expiratory volume per second, this breathing manoeuvre additionally allows the determination of the vital capacity.

Apart from the additional Tiffeneau test, the subjects were instructed to breathe different tidal volumes. In order to capture as much of the respiratory spectrum as possible, the subjects breathed shallowly by reducing their respiratory activity to a minimum. Subsequently, the subjects performed medium breaths, where they increased the tidal volume over the volume level of normal spontaneous breathing, but not to the extent of maximal breaths. Finally, they performed maximal breaths, where they inhaled and exhaled to the maximal possible. Each different breathing pattern was performed for approximately one minute, and before and after each breathing pattern, the subjects performed normal spontaneous breathing for about 30 s to recover and to prevent any kind of hyperventilation. 

**Figure 4:**
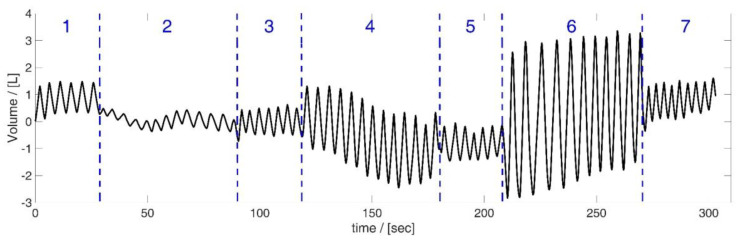
Volume data obtained by the spirometer during the respiratory manoeuvre, illustrated based on the data of subject 5. Manoeuvre: breathing different tidal volumes—periods of shallow (2), medium (4) and maximal breaths (6) between short periods of normal spontaneous breathing ((1), (3), (5) and (7)).

There was a pause between the Tiffeneau test and the manoeuvre. The length of the pause was determined by the subject itself—the manoeuvre was started when the subjects had fully recovered from Tiffeneau testing and felt ready to continue. However, the exact timing of the manoeuvre was not predetermined; it was dependent on the breathing rhythms of the subjects. Table 2 illustrates the details about the respiratory manoeuvre, and Figure 4 illustrates exemplarily the volumes measured by the spirometer during the manoeuvre. The total time of the measurement was about 5 min. 

To improve the optical capturing, the subjects were asked to place their arms on the rigid spirometer hold and to tie up long hair during the measurement, because long hair would cover MoCap markers, and therefore, would reduce the detection rate of the hidden MoCap markers.

### 2.4. Data Processing

All data processing in this study was based on the measured spatial positions of the 102 MoCap markers and their movement during breathing manoeuvres. The movement of the markers allowed for calculating changes in tilt angles in each MoCap marker position, movements along the principal component of the marker movement, occurring accelerations at the marker positions, the changes in distances between neighbouring markers and changes in upper body circumferences at different heights. 

However, prior to the individual calculations, an initial correction was made to the spatial marker positions obtained by the MoCap system. Each measured marker position was corrected so that the marker position was on the skin surface of the subject’s upper body and not in the centre of each MoCap marker (approximately 10 mm from the skin surface). This correction was done by calculating the imaginary centre of all markers in a specified height and subtracting the distance of the marker-midpoint to the skin surface from the distance of the centre of all markers to the marker-midpoint of the respective marker. (A detailed description of this performed correction procedure can be found in Laufer et al. [30]). The individual calculation methods of the different employed parameters are explained in the subsequent subsections.

#### 2.4.1. Movements along the Principal Component of the Marker Movement

Movements in the direction of the principal component of the marker movement of each marker were obtained. In a previous work (Laufer et al. [38]), it was evident that each marker moved predominantly along a particular line. Therefore, for each marker, this line (the main component of the marker motion) was identified using a singular value decomposition SVD. The subsequent projection of the spatial positions $\left(x\left(t_{k}\right),\;y\left(t_{k}\right),\;z\left(t_{k}\right)\right)$ on the principal component delivered the respiration induced movement *L* of each marker along the direction of its principal marker movement. The projection was done by using the *dot* function of MATLAB, which delivered the length of the projected vector *L_i_*, which was then used for further analysis. Details on the calculation of *L* can be obtained from Laufer et al. [38].

All linear trends and offsets were removed from the data by the *detrend*-function of MATLAB, which subtracts the best-fit line in the least-squares sense from the data. The movement Δ*L_i_* of each marker *i* (for 1 ≤ *i* ≤ 102) along its principal movement axis at each time point *t_ν_* with *t_1_* ≤ *t_ν_* ≤ *t_n_* is given by:

(1) $$\Delta L_{i}=\;{\left(\begin{array}{c} \Delta L_{t_{1}}\\ \vdots \\ \Delta L_{t_{n}} \end{array}\right)}_{i}$$

#### 2.4.2. Accelerations along the Principal Component of the Marker Movement

The second derivatives of *L_i_* delivered the desired accelerations *a_L,i_* of marker *i* (1 ≤ *i* ≤ 102) by:

(2) vL,i= dLi^dtaL,i=dvL,i^dt

where the “^” symbol denotes filtered, detrended values, and thus, vL,i^ is the filtered detrended velocity of marker *i* in the direction of the principal component of its movement. For filtering, a zero-phase low pass filter (*filtfilt* function of MATLAB) was used with a *PassbandFrequency* of 0.04, a *StopbandFrequency* of 0.1, a *PassbandRipple* of 0.5, and a *StopbandAttenuation* of 60 and the *detrend*-function removed all trends and offsets.

#### 2.4.3. Distances between Neighbouring Markers

By using strain gauges, expansions and distance changes can be measured on the surface of the upper body. Based on the MoCap data, the distances between all neighbouring markers were determined by the L_2_ norm function. The distance between MoCap marker *j* and MoCap marker *i* is calculated by:

(3) $$D_{m}=\|\left(x_{i},y_{i},z_{i}\right)-\left(x_{j},y_{j},z_{j}\right){\|}_{2}=\sqrt{{\left|x_{i}-x_{j}\right|}^{2}+{\left|y_{i}-y_{j}\right|}^{2}+{\left|z_{i}-z_{j}\right|}^{2}}$$

for all 1 ≤ *i* ≤ 102 and *j* as a direct neighbour of marker *i*. 

**Figure 5:**
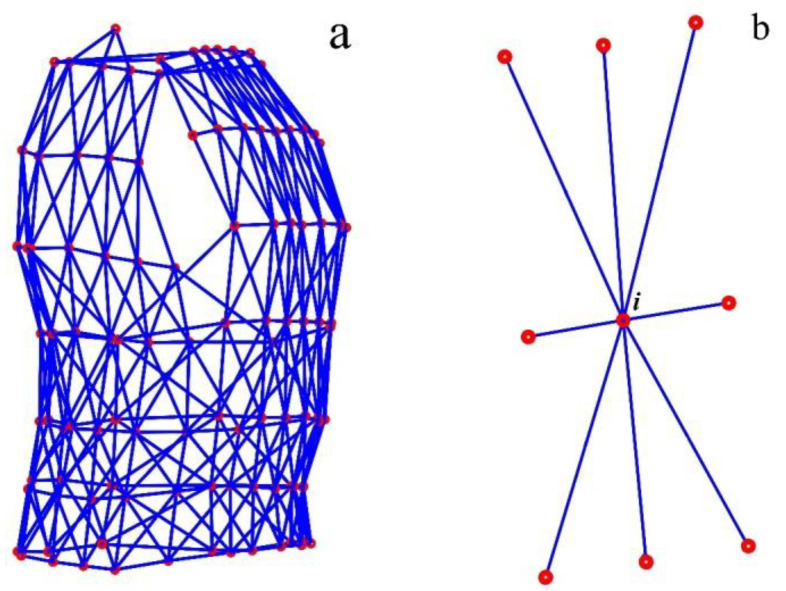
Distances between neighbouring MoCap markers—all 361 distances (blue) between MoCap markers (red), which were determined (**a**). The eight neighbouring MoCap markers of marker *i* in the middle of the illustrated marker-set (**b**).

The arrangement of the 102 MoCap markers on the compression shirt yielded *m* = 361 distance values, which were calculated and shown in Figure 5a. Figure 5b shows which MoCap markers were considered as direct neighbours of marker *i*. 

The *vecnorm* function of MATLAB was used for the calculation of distance values. Trends and offsets were also removed from the distances using the *detrend*-function of MATLAB. Thus, the distance between two neighbouring markers *i* and *j* was determined at each time point *t_ν_* with *t_1_* ≤ *t_ν_* ≤ *t_n_* of the measurement by:

(4) $$\Delta D_{m}=\;{\left(\begin{array}{c} \Delta D_{t_{1}}\\ \vdots \\ \Delta D_{t_{n}} \end{array}\right)}_{i,j}$$

#### 2.4.4. Circumferences

**Figure 6:**
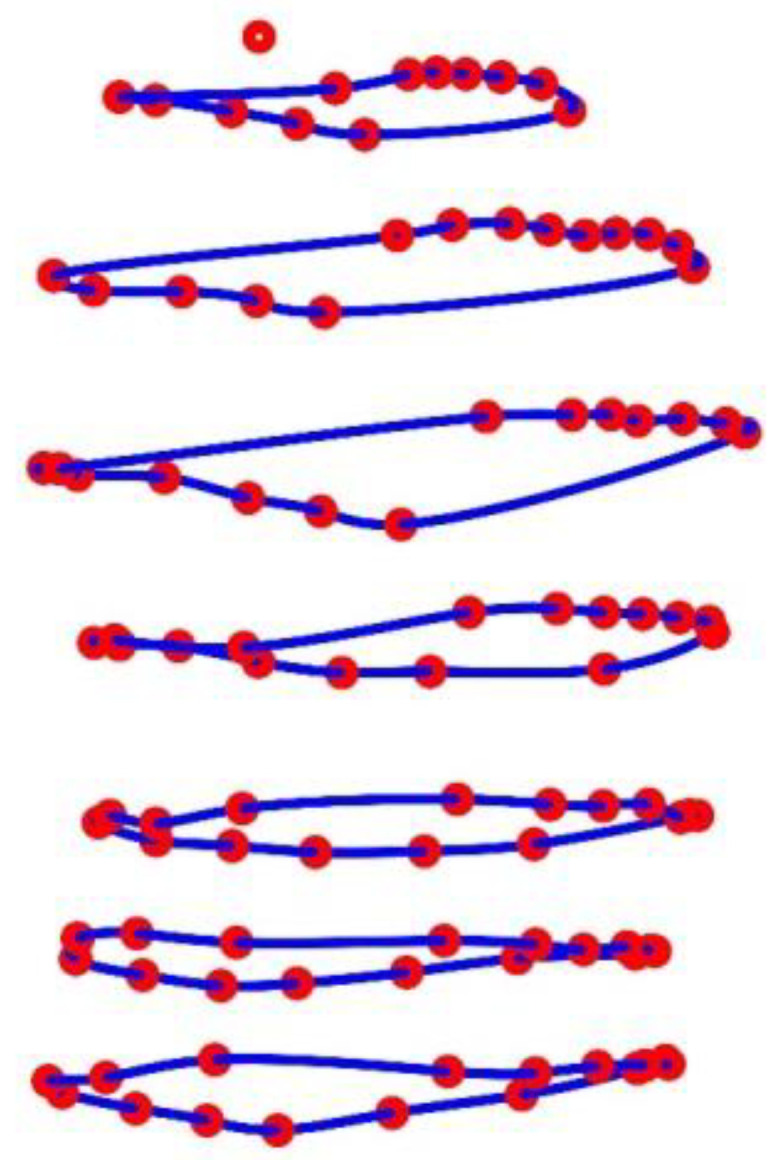
Illustration of the circumferences in 7 different heights at the upper body obtained via closed spline curves (blue) through the MoCap markers (red). The MoCap marker at the top is the reference marker.

Based on the MoCap markers, the circumferences in 7 different heights (excluding the height containing a single MoCap marker (reference point at C6)) at the upper body were finally calculated. Therefore, all MoCap markers in a height were connected via a closed spline curve, and the length of the spline curve was declared as the circumference *circ_k_* in height *k* (for all heights 1 ≤ *k* ≤ 7 with more than 1 MoCap marker) (see Figure 6). The *cscvn* function of MATLAB was employed for spline calculation, and the length of the spline curve was taken as the circumference, after removing trends and offsets (*detrend* function of MATLAB). Thus, the circumference was determined at each time point *t_ν_* with *t_1_* ≤ *t_ν_* ≤ *t_n_* of the measurement by:

(5) $$\Delta circ_{k}=\;{\left(\begin{array}{c} \Delta circ_{t_{1}}\\ \vdots \\ \Delta circ_{t_{n}} \end{array}\right)}_{k}$$

#### 2.4.5. Tilt Angles

**Figure 7:**
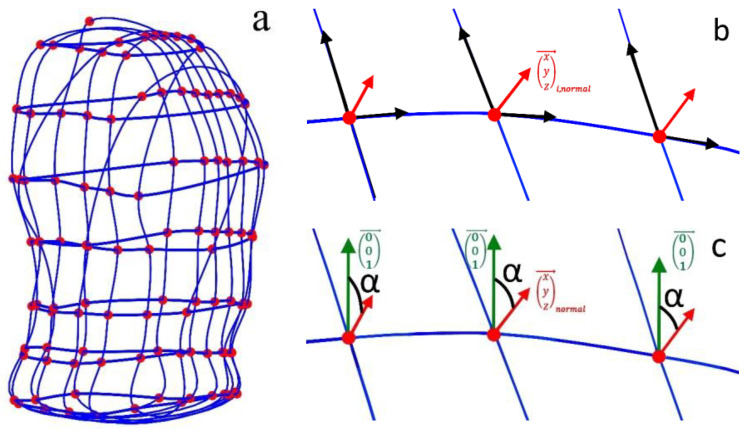
Illustration of the closed spline curves (blue) through horizontal and vertical marker sets, used to obtain tilt angles at the MoCap marker (red) positions (**a**) and the normal vectors (red arrows) to the tangent planes in the marker positions, spanned by the directional vectors—the derivatives of the spline curves (black arrows in (**b**)). The z-direction of the coordinate system is illustrated in green (**c**), and the obtained tilt angles α (black in (**c**)) are the angles between the z-direction and the normal vectors.

Tilt angles can be obtained by gyroscopes, implemented, e.g., in IMUs with 6 or 9 degrees of freedom (DOF). To obtain changes in tilt angles from the MoCap data, horizontal and vertical closed spline curves (Figure 7a) were used to determine tangential planes to the upper body surface at each MoCap marker position. In Figure 7b, the two direction vectors spanning the tangent plane are shown in black, and the normal vector, which is perpendicular to both direction vectors, is shown in red. The direction vectors are the derivatives of the horizontal and vertical spline curves in the MoCap marker positions, and the normal vectors ($\vec{x_{i,norm},y_{i,norm},z_{i,norm})}$ are obtained by the cross product of these two direction vectors (*cross* function of MATLAB). Afterwards, the tilt angles refer to the z-axis *α_tilt,i,z_* (illustrated as green vectors in Figure 7c) and are obtained for all MoCap markers (1 ≤ *i* ≤ 102) at each time point *t_ν_* with *t_1_* ≤ *t_ν_* ≤ *t_n_* by:

(6) $$\alpha_{tilt,i,z}=180-arccos\left(\frac{\vec{\left(\begin{array}{c} 0\\ 0\\ 1 \end{array}\right)}\cdot \vec{{\left(\begin{array}{c} x_{i,norm}\\ y_{i,norm}\\ z_{i,norm} \end{array}\right)}_{i}}}{\|\vec{{\left(\begin{array}{c} x_{i,norm}\\ y_{i,norm}\\ z_{i,norm} \end{array}\right)}_{i}}{\|}_{2}}\right)\cdot \frac{180}{\pi }$$

Thus, for each marker *i* (for 1 ≤ *i* ≤ 102), the tilt angle was obtained at each time point *t_ν_* with *t_1_* ≤ *t_ν_* ≤ *t_n_* of the measurement by the vector:

(7) $${\vec{\Delta \alpha_{tilt}}}_{i}={\left(\begin{array}{c} \Delta \alpha_{tilt,t_{1}}\\ \vdots \\ \Delta \alpha_{tilt,t_{n}} \end{array}\right)}_{i}$$

To remove any linear trends and offsets from the data, each tilt angle vector was corrected using the *detrend*-function of MATLAB.

#### 2.4.6. Ranges and Correlations

In order to obtain the ranges of the individual parameters that can occur during breathing, the changes of the analysed parameter for each marker during all breaths were analysed. Breath by breath, the change of the particular parameter Δ*P* caused by exhalation and inhalation was determined by:

(8) $$\Delta P_{i}\left(t^{*}\right)=\|\max\left(P_{i}\left(t_{\nu }{}^{*}\right)\right)-\min\left(P_{i}\left(t_{\nu }{}^{*}\right)\right){\|}_{2}$$

for all breaths *b* with $t_{\nu }{}^{*}={\left[t_{1,\nu }^{*},t_{2,\nu }^{*}\right]}_{b}$ and *t*_1_ < $\left[t_{1,\nu }^{*},t_{2,\nu }^{*}\right]$ *< t_n_*, where $t_{j}{}^{*}$ is the timeframe of a single breath in the specified breathing pattern. Each single breath *b* has the timeframe $t_{\nu }{}^{*}$ in the specified breathing pattern with $t_{\nu }{}^{*}={\left[t_{1,\nu }^{*},t_{2,\nu }^{*}\right]}_{b}$ and *t*_1_ < $\left[t_{1,\nu }^{*},t_{2,\nu }^{*}\right]$ *< t_n_*.

This was performed for all the particular parameters *P* in all MoCap marker positions *i* with 1 ≤ *i* ≤ 102, analysing distances for all *m* with 1 ≤ *m* ≤ 361 and when investigating circumferences for all *k* with 1 ≤ k ≤ 7. 

The areas of interest for determining the ranges are the maximum changes during maximum breaths and shallow breaths. Smaller maximal changes are expected during shallow breaths, while it can be assumed that the largest maximum changes are detectable during maximal breaths. The determination of the maximum changes is the main focus here, since the signal-to-noise ratio of the sensors used is highest during maximum changes, which improves measurement results.

(9) $$Range_{\mathrm{maximal}\mathrm{breaths}}=\underset{t}{\max}\;\left\{\Delta P\left(t^{*}\right):\;\mathrm{over}\;\mathrm{all}\;\mathrm{maximal}\;\mathrm{breaths}\right\}$$

(10) $$Range_{\mathrm{shallow}\;\mathrm{breathing}}=\underset{t}{\max}\;\left\{\Delta P\left(t^{*}\right):\;\;\mathrm{over}\;\mathrm{all}\;\mathrm{breaths}\;\mathrm{in}\;\mathrm{shallow}\;\mathrm{breathing}\right\}$$

Additionally, the correlations between the parameters and the volume signal of the spirometer *V_spiro_* were identified by the Pearson correlation coefficients $\;R_{P,V_{spiro}}$:

(11) $$R_{P,V_{spiro}}=\frac{\sum_{t=t_{1}}^{t_{n}}\left(P\left(t\right)-\bar{P}\right)\left(V_{spiro}\left(t\right)-\bar{V_{spiro}}\right)}{\sqrt{\sum_{t=t_{1}}^{t_{n}}{\left(P\left(t\right)-\bar{P}\right)}^{2}}\sqrt{\sum_{t=t_{1}}^{t_{n}}{\left(V_{spiro}\left(t\right)-\bar{V_{spiro}}\right)}^{2}}}$$

where *P* is the examined parameter (e.g., circumferences, tilt angles or others) at each time point *t* of the selected measurement interval.

The *corrcoef*-function of MATLAB was utilised to obtain the desired correlation coefficients, and the corresponding *p*-values were checked if they were less than 0.05 (the result can be considered statistically significant).

The determined measurement ranges show the order of magnitude of the specified parameters, which cover the measurement range from clinical application to home care and sports activities, and thus represent the measurement ranges of these parameters in a smart shirt that are available for respiration analysis.

## 3. Results

**Figure 8:**
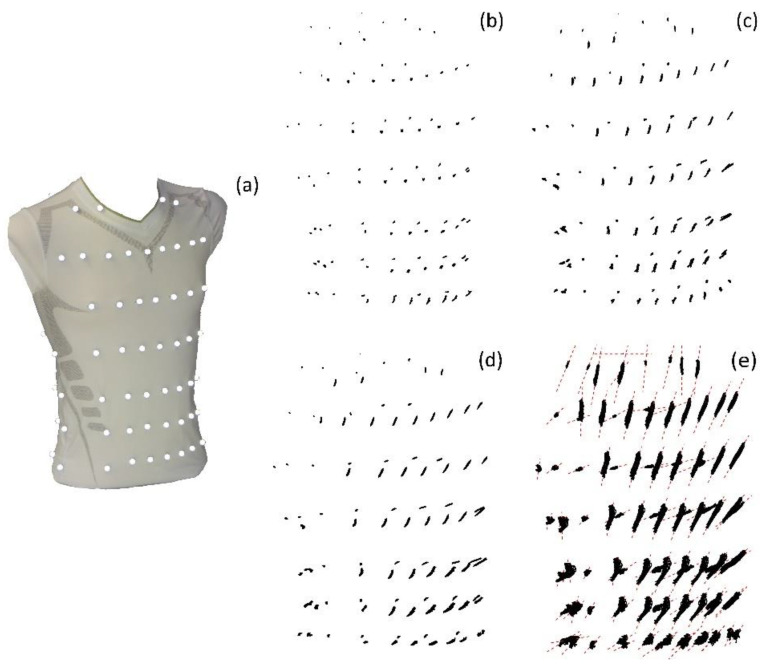
Spatial movement of the MoCap markers on the compression shirt (**a**) during shallow breathing (**b**), normal spontaneous breathing (**c**), medium breaths (**d**) and maximal breaths (**e**), illustrated based on the data of subject 5. The MoCap markers move predominantly on a specific line, which are illustrated in (**e**) as red dashed lines.

The spatial movements of the MoCap markers are illustrated in Figure 8 for shallow breathing (b), normal spontaneous breathing (c), medium breaths (d) and maximal breaths (d). The corresponding compression shirt is shown in Figure 8a.

The vital capacities obtained by the Tiffeneau test were compared by the maximal vital capacity during breathing pattern 6 (maximal breaths). The results of the comparison are illustrated in Table 3.

In Table 4, the maximal measured ranges and correlations to *V_spiro_* during shallow breathing and during maximal breaths of the analysed movement parameters are provided. All values are given in the units of the respective parameter. In addition, the mean and standard deviation of the parameters across all subjects are given.

In the following figures, the representation of the ranges of the individual motion parameters and their correlations with the spirometer volume *V_spiro_* are shown graphically.

### 3.1. Movements

**Figure 9:**
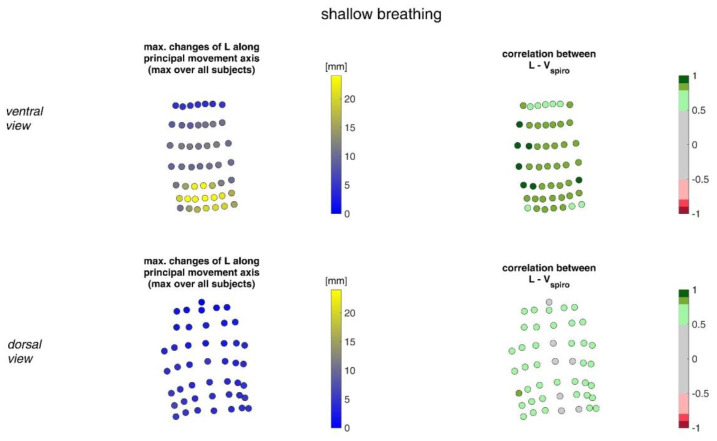
The maximal changes of the movements of MoCap markers along their principal movement axis during shallow breathing (mean over all subjects (**left**)) and the mean corresponding correlation of these changes with spirometer volume *V_spiro_* of all subjects during shallow breathing are shown (**right**). (**Top**-ventral view, **bottom**—dorsal view).

Figure 9 illustrates the movement ranges of the MoCap markers along their principal movement axis and their correlation with *V_spiro_* during shallow breathing.

**Figure 10:**
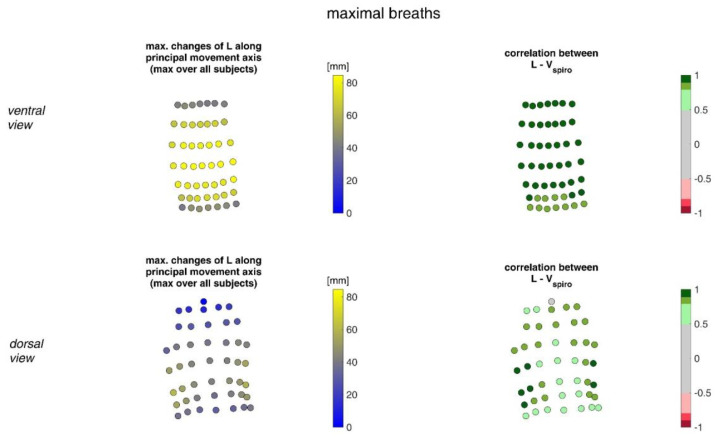
Illustration of the movement of MoCap markers at the upper body along their principal movement axis. The largest movements amongst all subjects during maximal breaths (**left**) and the mean corresponding correlation of maximal movements with spirometer volume *V_spiro_* of all subjects during maximal breaths is shown (**right**). (**Top**—ventral view, **bottom**—dorsal view).

Figure 10 shows the movement ranges of the MoCap markers along their principal movement axis during maximal breaths and their correlation of the movement with *V_spiro_*.

### 3.2. Accelerations

**Figure 11:**
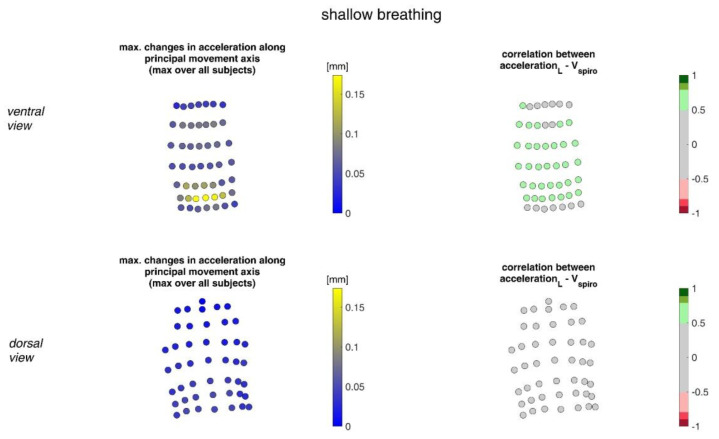
The maximal accelerations of MoCap markers along their main movement axis during shallow breathing (mean over all subjects (**left**)) and the mean corresponding correlation of these changes with spirometer volume *V_spiro_* of all subjects during shallow breathing are shown (**right**). (**Top**—ventral view, **bottom**—dorsal view).

In Figure 11, the acceleration ranges of the MoCap markers along their principal movement axis and their correlation with *V_spiro_* during shallow breathing are displayed.

**Figure 12:**
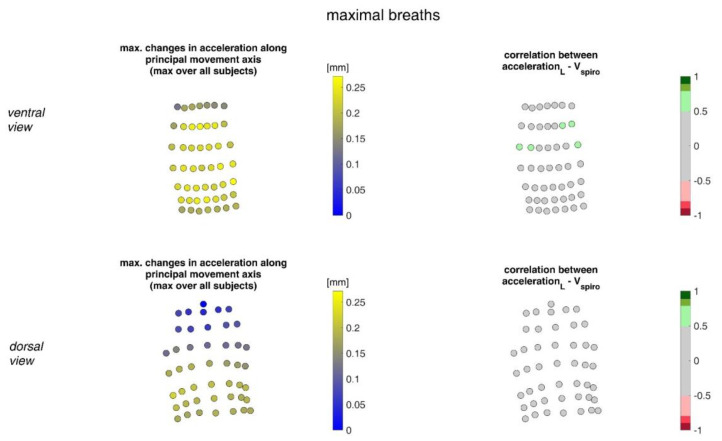
Illustration of the accelerations of MoCap markers along their main movement axis. The largest accelerations amongst all subjects (**left**) during maximal breaths and the mean corresponding correlation of maximal accelerations with spirometer volume *V_spiro_* of all subjects during maximal breaths are shown (**right**). (**Top**—ventral view, **bottom**—dorsal view).

Figure 12 illustrates the acceleration ranges of the MoCap markers along their principal movement axis and their correlation with *V_spiro_* during maximal breaths.

### 3.3. Distances

**Figure 13:**
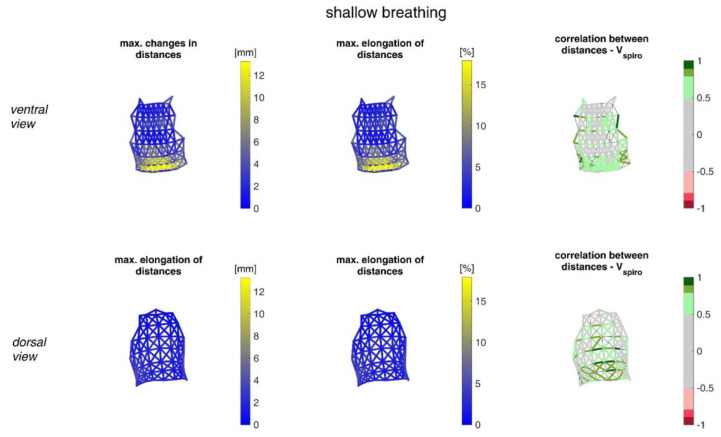
Illustration of the maximal distance changes amongst all subjects during shallow breathing (**left**), the corresponding distance elongation of all subjects (**center**), and the mean corresponding correlation of distance changes with *V_spiro_* of all subjects during shallow breathing (**right**). (**Top**—ventral view, **bottom**—dorsal view). The dotted lines are distances, which do not fulfil (*p* < 0.05).

The ranges of the distance changes between neighbouring MoCap markers and their correlation with the spirometer volume *V_spiro_* are shown in Figure 13 for shallow breathing.

**Figure 14:**
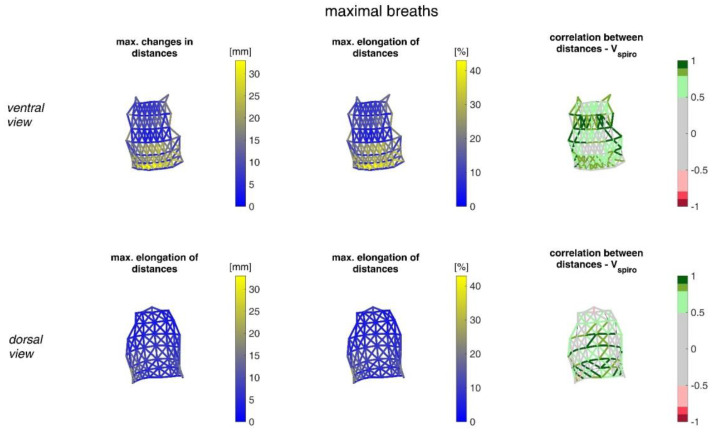
Illustration of the distance changes and elongations between MoCap markers at the upper body. The largest distance changes amongst all subjects during maximal breaths (**left**), the maximal distance elongation (**center**), and the mean corresponding correlation of maximal distances with *V_spiro_* of all subjects during maximal breaths are shown (**right**). (**Top**—ventral view, **bottom**—dorsal view). The dotted lines are the distances, which do not fulfil the requirement (*p* < 0.05).

Figure 14 illustrates distance changes between neighbouring MoCap markers and their correlation with *V_spiro_* during maximal breaths.

### 3.4. Circumferences

**Figure 15:**
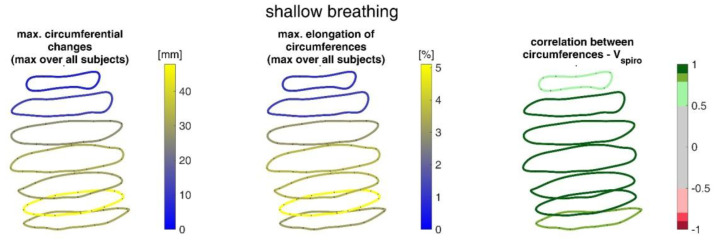
Illustration of the maximal circumference changes amongst all subjects during shallow breathing (**left**), the corresponding circumference elongation (**center**), and the mean corresponding correlation of circumferences with spirometer volume *V_spiro_* of all subjects during shallow breathing are shown (**right**).

Figure 15 shows the ranges of circumferential changes of the upper body circumferences and their correlation with the spirometer volume *V_spiro_* during shallow breathing.

**Figure 16:**
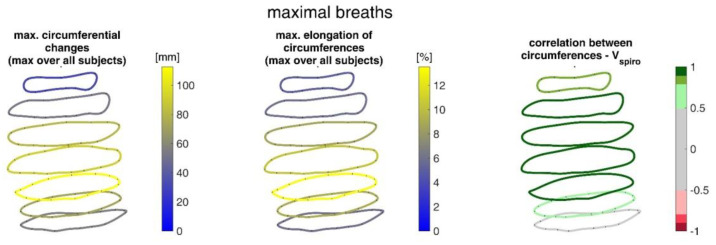
Illustration of the circumferences at seven different heights on the upper body determined via closed spline curves by the MoCap markers. The largest circumference changes amongst all subjects during maximal breaths (**left**), the maximal corresponding circumference elongation (**center**), and the mean corresponding correlation of circumferences with spirometer volume *V_spiro_* of all subjects during maximal breaths are shown (**right**).

The ranges of circumferential changes of the upper body circumferences and their correlation with *V_spiro_* during maximal breaths are illustrated in Figure 16.

### 3.5. Tilt Angles

**Figure 17:**
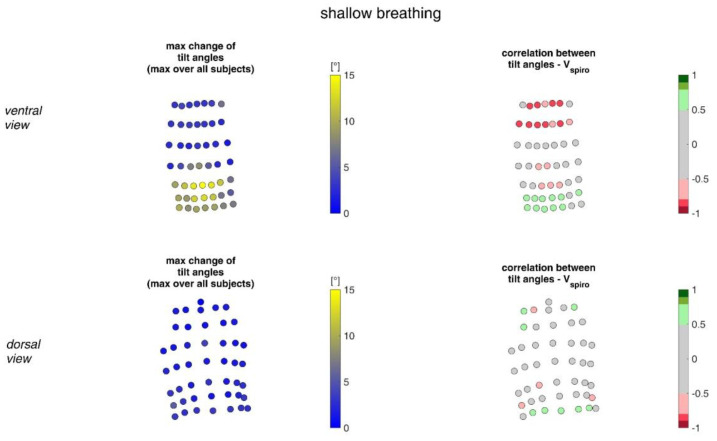
The maximal tilt angle changes amongst all subjects during shallow breathing (**left**) and the corresponding correlation with the spirometer volume *V_spiro_* of all subjects during shallow breathing are shown (**right**). (**Top**—ventral view, **bottom**—dorsal view).

Figure 17 shows the ranges of tilt angle changes and their correlation with *V_spiro_* during shallow breathing.

**Figure 18:**
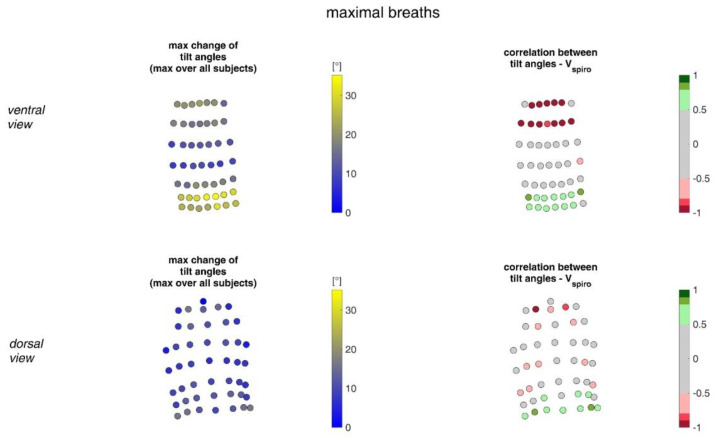
Illustration of the changes of tilt angles at the MoCap marker position at the upper body. The largest tilt angle changes amongst all subjects during maximal breaths (**left**) and the mean corresponding correlation of maximal tilt angle changes with spirometer volume *V_spiro_* of all subjects during maximal breaths are shown (**right**). (**Top**—ventral view, **bottom**—dorsal view).

Figure 18 shows the ranges of tilt angle changes and their correlation with *V_spiro_* during maximal breaths.

## 4. Discussion

Despite high initial costs, the clinical application of the OEP shows that there is a need for respiratory flow measurement alternatives. However, the costs of the MoCap system limit applications to those where respiration should not be compromised in any way, such as the respiratory monitoring of premature infants. Existing wearable systems such as the Hexoskin system [31] have recently been scrutinised in different studies and show reliable results for heart rate and respiratory rate [33,34]. However, the accuracy of the Hexoskin system estimation of respiratory volumes does not exceed the threshold required for clinical applications, and further research is still ongoing. 

This study was conducted to support further development of smart shirts measuring tidal volumes by investigating respiration-induced motion by compiling motion capture information from multiple sensors on the upper body. Data from a MoCap system, with measurement accuracy in the sub-millimetre range, were captured, various motion parameters were determined and their potential contribution to respiratory volume estimation investigated. For surface motion measurement precision, the MoCap system provides accuracy in the sub-millimetre range. Other sensors might have higher measurement accuracy under ideal conditions, but this can decrease in specific applications—usually due to the design of the measurement device or other circumstances.

In order to obtain the most accurate measurement results, non-respiration induced movements of the upper body were almost eliminated during the measurement by fixing the spirometer on a stable holder at the height of the subjects’ mouths. Thus, the subjects could only move their heads to a limited extent during the experiment. Another advantage of fixing the spirometer was that the subjects could rest their hands on this holder, which significantly improved their comfort during the measurement, and the subjects’ arms were in a position that did not obscure the detection of the MoCap markers. This allowed stable measurement of upper body movements during different breathing patterns, which were almost exclusively limited to respiration-induced movements.

This measurement is based on the movements of MoCap markers. By using the spatial positions of the MoCap markers to calculate the volume enclosed by the markers (*alphaShape* function of MATLAB), deviations from the spirometer volume occur, especially for larger breaths [39], probably caused by pressure-related compressions of the air in the thorax, while these compressions do not affect the flow measurement of the spirometer. Hence, it could occur that the deviations are to some extent transferred to the investigated parameters.

The breathing manoeuvre performed (Table 2) ensured that almost the entire spectrum of tidal volumes was captured during breathing—from shallow breathing to maximal breath. Shallow breathing, in which subjects try to inhale as little air as possible, is usually associated with minimal upper body breathing movements and can be used to analyse the minimal ranges of parameters. These shallow breaths may indicate patients with specific respiratory diseases. The maximum parameter changes during the maximum breaths can be observed as well. These maximal breaths may be more relevant to healthy individuals undertaking aerobic exercise. However, with respect to accelerations that occur, shallow or maximal breathing patterns do not necessarily imply minimal and maximal accelerations of surface motion. In particular, the respiratory rate is often subconsciously increased during shallow breathing, which can lead to higher acceleration values even with lower movement amplitudes. However, in most cases, the increase in breathing frequency was minor, therefore, this issue could be neglected during evaluation. 

Prior to the measurement, the subjects underwent a Tiffeneau test to determine their vital capacity. In addition, the vital capacity was determined during maximum breathing in the manoeuvre (breathing pattern six). The comparison shows that some subjects almost reached the vital capacity of the Tiffeneau test during maximal breaths (subjects 1, 9 and 13), while in others (subjects 7 and 12) a discrepancy of up to 48% was found (Table 3). This discrepancy implies how much the results can be dependent on the motivation of the subjects and on the trained personnel. When performing the Tiffeneau test, the subjects were strongly motivated by the supervisor, whereas during the manoeuvre they were only instructed to breathe maximally several times.

The measurements performed provided a number of metrics that captured the motion of the upper body during shallow and maximal breathing (Table 4). In particular, circumferential changes of up to 113 mm (13.5% of end-expiratory circumference) occurred during maximum breaths (Figure 15). Here, the largest circumferential changes occurred between the level of the thoracic vertebra T4 and the lumbar vertebra L1. Changes in the spatial position at the upper body, i.e., movements along the major axis of motion of the MoCap markers, occurred up to 85 mm anteriorly (ventrally) between the level of T1 and L1 (Figure 7), and inclination angles changed by up to 36 degrees in the ventral region (Figure 17). Distance changes between adjacent MoCap markers were a maximum of 33 mm, corresponding to a percentage expansion of up to 43% (Figure 13). The largest proportional distance changes occurred predominantly in the lateral and inferior dorsal regions. Maximal accelerations detected at the MoCap markers were 0.27 mm/s^2^. The acceleration values captured represent the design range of IMU sensors in smart shirt applications. However, the maximum accelerations that occur are below the range of the most inexpensive IMUs on the market, resulting in low signal-to-noise ratios [22]. This problem is exacerbated for smaller breaths.

During shallow breathing, the maximum circumferential changes in the order of 5.1% were measured, which corresponds to an extension of about 48 mm (Figure 16). In contrast to maximal breaths, larger circumferential changes occurred toward the abdominal region between the levels of the thoracic vertebra T4 to the lumbar vertebra L3. The greatest changes in spatial positions occurred in the abdomen, anteriorly between T4 and L1. The greatest elongations between markers occurred predominantly laterally and in the abdominal region. The tilt angles, with respect to the vertical, also changed most predominantly in the lower abdominal region, up to 15 degrees (Figure 18). 

**Figure 19:**
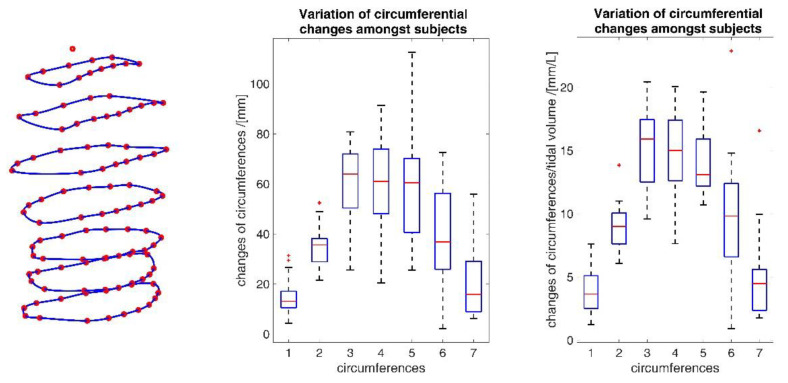
Inter-individual variability of circumferential changes amongst all subjects during maximal breathing (**middle**) and variability of circumferences considering different inspired tidal volumes (**right**). The blue numbers identify the circumferences as shown (**left**). The deployed box and whisker plot illustrates the minimum value, 25% percentile, median, 75% percentile, the maximum value and the red + signs denote outliers.

The comparatively high standard deviations observed in parameters during shallow breathing (Table 4) indicate an inter- and intra-individual variability of the measurements of different subjects and, therefore, changes in the movement parameters can vary considerably from person to person. Different tendencies to abdominal or thoracic breathing or different breathing frequencies do not exclusively influence the results; in addition, different tidal volumes affected the outcomes. In Figure 19, the data variability of the individual subjects is exemplarily shown in the circumferential changes. Since the different tidal volumes respired by the subjects (Table 3) may be a significant factor for the high standard deviation, the variation of the data with respect to the tidal volumes is also shown. This indicates that the different tidal volumes certainly have an influence on the variability, but that the variability also depends on other factors, such as different tendencies to abdominal or chest breathing. 

However, it could be observed across subjects that during shallow breathing, subjects tended to abdominal breath and while during maximal breathing, subjects used the entire capacity of the lungs and maximised both abdominal and thoracic breathing. Thus, during maximal breathing, the tendency to recruit the thorax or abdomen was mitigated in participants, and there was greater inter-participant consistency in motion for maximal breathing.

This study investigates the individual correlation of each parameter with the spirometer volume *V_spiro_*. In general, these correlations were higher at maximal breathing volumes than at low breathing volumes. This could be due to the fact that larger amplitudes and lower respiratory frequencies occurred during larger respiratory volumes, and thus there were almost no fast changes of the parameters, which usually implies a better signal-to-noise ratio.

The upper body circumferences were well correlated to breath volume (Figure 15 and Figure 16). However, a simpler approach to capture this outcome was published as early as 1965 by Agostoni et al. [31]. The present study supports these results, as the highest correlations with spirometer volume of all measured data were found for mean upper body circumference between the level of thoracic vertebra T4 and lumbar vertebra L1. Correlations of 0.97 were found for maximal breaths (Figure 15 and Table 4), while the highest correlations for shallow breaths ranged from 0.7 to 0.9 (Figure 16).

The highest correlations between *V_Spiro_* and distance changes between markers (R ≈ 0.97) were found in the lower dorsal region and laterally on the torso (Figure 13 and Figure 14). Tilt angle changes correlated with *V_spiro_* predominantly ventral in the higher chest area and in the lower abdominal part (Figure 17 and Figure 18). As expected, the dorsal tilt angle changes were only partially correlated with the spirometer volume. Accelerations themselves did not correlate well with the volume signal. In practice, acceleration data from surface mounted IMU sensors would most likely undergo a two-fold integration to obtain the spatial positions, which are then correlated to inspired volume changes. However, the raw acceleration observed in the spontaneous breathing data (Figure 11 and Figure 12) does not appear to provide a useful correlation to inspired volume in this study.

It should be noted that some of the parameters of the markers carrying system information were highly correlated with each other. In particular, especially the markers at the thorax generally moved in unison and thus, simply selecting the markers with the highest individual correlations may lack information uniqueness and may not represent the highest information content with respect to respiratory volumes possible [38]. Therefore, further increasing the number of sensors could have a very minor effect on the information content of the overall measurement system. Thus, the identification of a sensor set carrying a maximum of respiratory information is only supported by this and not carried over.

An appropriate selection of sensors and their locations in a smart shirt approach must be optimised to enable accurate and precise volume estimation from surface motion. This article presents the ranges and tidal volume correlations for a number of metrics that could be obtained with common sensors. The correlations with respiratory volume provide an indication of how much respiratory information the corresponding metric carries. If sensors are placed at a location in a smart shirt where the corresponding metric has little correlation with respiratory volume, that sensor adds little additional respiratory information to the measurement system. Therefore, the knowledge of correlation is critical to the selection, placement and implementation of sensors in the smart shirt. To the authors’ knowledge, no such compilation of knowledge exists in the current literature. 

One more potential diagnostic and clinically relevant aspect would be to check symmetry of the upper body motions using a smarts shirt. A smart shirt with appropriate sensor technology could reveal symmetries or asymmetries of the movement of the upper body and could be used to diagnose associated diseases. In particular, asymmetric upper body movement patterns can help to diagnose diseases such as pneumothorax, broken ribs, unilateral lung disease, severe atelectasis, or emphysema [40,41]. It is reasonable to suspect that even with circumferential measurements alone, a clever arrangement of optical encoders could determine symmetric properties of upper body motion and possibly provide further insight into diagnosis. Hence, in typical breathing, chest and abdomen motions are either both expanding, or both contracting. In the case of diaphragmatic dysfunction, the chest and abdomen motions occur in opposite directions, which is called the Hoover sign [42,43,44]. As such, if validated in clinical research, it may be possible to diagnose diaphragmatic dysfunction with only circumferential measurements. 

Limitations in these measurements were mainly due to the fact that the size of the shirt introduced some error. The variance in fit of the compression shirt across participants with distinct morphology imply that there would be slightly different marker locations. However, care was taken during fitting to ensure the certain markers were located on specific physiological features, and thus mitigating this concern.

Further limitations were that during maximal breathing, it was likely that the shirt moved in relation to the skin surface [45]. However, this would generally also occur with a smart shirt, and thus, represent a typical systematic error that seems difficult to avoid without irritating the participant. In particular, it may be possible to reduce the shirt-to-skin movement with a very tight fit of the smart shirt and/or an adhesive or high friction inner-fabric to adhere better to the skin. However, the potential benefit of such an approach may not lead to improved results. In particular, there are multiple tissue interfaces between the alveolar and the skin, which shift with breathing activities. Adding a further layer of relative motion at the skin-shirt interface hardly seems like a confounding factor. Furthermore, since the correlations shown in Table 4 are of sufficient strength to imply a precise estimation of *V_spiro_*, it may not be profitable to pursue higher precision for increased cost or discomfort. In addition, measurements with more subjects of different ages and with different body shapes could confirm the results of this study and give a better insight into the systematic nature of the changes in the parameters studied. In particular, most of the participants were male (13/16), young adults (13/16 ≤ 30) and in the healthy BMI range (15/16). As the study examined changes in measurable parameters on the upper body and by varying fitness levels of the subjects, as indicated by vital capacities ranging from 3.1 L even up to 6.8 L, a very wide range of surface motion was obtained. Measurements with more subjects of different ages and with different body shapes would be advantageous.

Furthermore, a study with subjects suffering from lung disease, e.g., patients with chronic obstructive pulmonary disease or cystic fibrosis, could show the applicability and the special requirements of a smart shirt for this field of application.

This research offers a survey of measurement ranges and correlations to determine tidal volumes with common sensors. It is an important contribution in the field of smart clothing/wearables, as this work shows the required measurement ranges and the expected correlation to respiratory volume depending on the placement of the sensors on the upper body and thus significantly supports the development of a smart shirt for respiratory volume determination and breath analysis.

## 5. Conclusions

This study provides a basis for the development of a smart shirt to estimate respiratory volume. The measurements obtained may help in the selection of the type and optimal location of candidate sensors that would allow tidal volumes to be measured with sufficient accuracy for clinical applications. Such a smart shirt could expand the availability of respiratory diagnostics and would allow more convenient and long-term measurement of respiratory parameters in home care or in the clinic.

## Figures and Tables

**Table 1:** Details of the participants.

Subject	Height/ [m]	Weight/ [kg]	BMI/ [kg/m^2^]	Age/ [Years]	Gender	Vital Capacity/ [L]
1	1.84	75	22.15	18	male	4.76
2	1.72	65	21.97	19	female	3.87
3	1.70	56	19.38	26	male	3.99
4	1.67	57	20.44	18	female	3.12
5	1.83	78	23.29	30	male	6.47
6	1.75	70	22.86	32	male	5.39
7	1.79	75	23.41	53	male	5.12
8	1.74	63	20.81	20	male	4.94
9	1.70	68	23.53	24	male	4.41
10	1.82	73	22.04	30	male	6.83
11	1.74	81	26.75	31	male	4.99
12	1.73	67	22.39	19	male	4.95
13	1.71	60	20.52	23	male	4.09
14	1.68	66	23.38	21	female	4.45
15	1.88	75	21.22	20	male	5.93
16	1.83	82	24.49	28	male	6.75

**Table 2:** Respiratory manoeuvre.

Pattern Number	Duration /[sec]	Breathing Pattern
1	30	spontaneous breathing (normal)
2	60	shallow breathing
3	30	spontaneous breathing (normal)
4	60	medium breaths
5	30	spontaneous breathing (normal)
6	60	maximal breaths
7	30	spontaneous breathing (normal)

**Table 3:** Vital capacities obtained by the Tiffeneau Test compared to the maximal vital capacities obtained during maximal breaths.

Subject	Vital Capacity VC by Tiffeneau Test/[L]	Max. Vital Capacity VC during Max. Breaths/[L]	Difference/ [L]	Difference/ [%]
1	4.76	4.72	0.04	0.8
2	3.87	3.50	0.37	9.6
3	3.99	3.34	0.65	16.3
4	3.12	2.02	1.10	35.3
5	6.47	5.74	0.73	11.3
6	5.39	3.75	1.64	30.4
7	5.12	2.67	2.45	47.9
8	4.94	3.87	1.07	21.7
9	4.41	4.22	0.19	4.3
10	6.83	5.59	1.24	18.2
11	4.99	3.35	1.64	32.9
12	4.95	2.87	2.08	42.0
13	4.09	3.83	0.26	6.4
14	4.45	3.80	0.65	14.6
15	5.93	5.20	0.73	12.3
16	6.75	5.62	1.13	16.7

**Table 4:** Ranges and correlations of the determined movement parameters.

Parameter	Unit	Shallow Breathing		Maximal Breaths	
		Max. Range (Mean/std)	Max. R^2^	Max. Range (Mean/std)	Max. R^2^
Spatial movements in the main direction of the MoCap marker movement	mm	24.1 (8.2/6.1)	0.91	84.6 (52.9/19.5)	0.97
Accelerations along the main direction of each MoCap marker movement	mm/s^2^	0.17 (0.05/0.03)	0.53	0.27 (0.18/0.06)	0.49
Δ distances between neighbouring markers	mm	13.2 (2.9/2.8)	0.95	33.0 (9.6/6.9)	0.97
Elongation distances	%	17.9 (3.9/3.8)	0.95	43.0 (12.7/9.0)	0.97
Absolute changes of body circumferences	mm	47.8 (26.4/14.1)	0.93	112.7 (71.0/27.0)	0.97
Elongation circumferences	%	5.1 (2.9/1.4)	0.93	13.5 (8.4/3.0)	0.97
Δ α_tilt_	°	15.0 (4.0/3.4)	0.88	35.2 (14.1/7.1)	0.96

## Data Availability

The data presented in this study are available on request from the corresponding author.

## Abbreviations

The following abbreviations are used in this manuscript:

α	Tilt angle
CiMeD	Circumference measurement device
Cx	Cervical vertebrae x (1 ≤ x ≤ 7)
DOF	Degrees of freedom
IMU	Inertial measurement unit
L	Length along main movement axis
Lx	Lumbar vertebrae x (1 ≤ x ≤ 5)
MoCap	Motion capture system
OEP	Optoelectronic plethysmography
P	Examined parameter
*p*	*p*-value
R	Pearson correlation coefficient
RIP	Respiratory inductance plethysmograph
std	Standard deviation
Tx	Thoracic vertebrae x (1 ≤ x ≤ 12)
VC	Vital capacity
*V_spiro_*	Volume obtained by the spirometer

## References

[1] Konno K., Mead J. (1967). Measurement of the Separate Volume Changes of Rib Cage and Abdomen during Breathing. J. Appl. Physiol..

[2] Parreira V.F., Vieira D.S., Myrrha M.A., Pessoa I.M., Lage S.M., Britto R.R. (2012). Optoelectronic Plethysmography: A Review of the Literature. Braz. J. Phys. Ther..

[3] Massaroni C., Carraro E., Vianello A., Miccinilli S., Morrone M., Levai I.K., Schena E., Saccomandi P., Sterzi S., Dickinson J.W. (2017). Optoelectronic Plethysmography in Clinical Practice and Research: A Review. Respiration.

[4] Heyde C., Mahler H., Roecker K., Gollhofer A. (2015). A Wearable Respiratory Monitoring Device–the between-Days Variability of Calibration. Int. J. Sport. Med..

[5] Heyde C., Leutheuser H., Eskofier B., Roecker K., Gollhofer A. (2014). Respiratory Inductance Plethysmography-a Rationale for Validity during Exercise. Med. Sci. Sport. Exerc..

[6] Miller M.R., Hankinson J., Brusasco V., Burgos F., Casaburi R., Coates A., Crapo R., Enright P., van der Grinten C.P.M., Gustafsson P. (2005). Standardisation of Spirometry. Eur. Respir. J..

[7] Hayes D.J., Kraman S.S. (2009). The Physiologic Basis of Spirometry. Respir. Care.

[8] Coates A.L., Peslin R., Rodenstein D., Stocks J. (1997). Measurement of Lung Volumes by Plethysmography. Eur. Respir. J..

[9] Criée C.P., Sorichter S., Smith H.J., Kardos P., Merget R., Heise D., Berdel D., Köhler D., Magnussen H., Marek W. (2011). Body Plethysmography--Its Principles and Clinical Use. Respir. Med..

[10] Askanazi J., Silverberg P.A., Foster R.J., Hyman A.I., Milic-Emili J., Kinney J.M. (1980). Effects of Respiratory Apparatus on Breathing Pattern. J. Appl. Physiol. Respir. Environ. Exerc. Physiol..

[11] Gilbert R., Auchincloss J.H.J., Brodsky J., Boden W. (1972). Changes in Tidal Volume, Frequency, and Ventilation Induced by Their Measurement. J. Appl. Physiol..

[12] Sibson F. (1848). On the Movements of Respiration in Disease, and on the Use of a Chest-Measurer. Med. Chir. Trans..

[13] WADE O.L. (1954). Movements of the Thoracic Cage and Diaphragm in Respiration. J. Physiol..

[14] Kaneko H., Horie J. (2012). Breathing Movements of the Chest and Abdominal Wall in Healthy Subjects. Respir. Care.

[15] Mannée D., de Jongh F., van Helvoort H. (2021). The Accuracy of Tidal Volume Measured With a Smart Shirt During Tasks of Daily Living in Healthy Subjects: Cross-Sectional Study. JMIR Form. Res..

[16] Aliverti A. (2017). Wearable Technology: Role in Respiratory Health and Disease. Breathe.

[17] Monaco V., Stefanini C. (2021). Assessing the Tidal Volume through Wearables: A Scoping Review. Sensors.

[18] Khundaqji H., Hing W., Furness J., Climstein M. (2020). Smart Shirts for Monitoring Physiological Parameters: Scoping Review. JMIR Mhealth Uhealth.

[19] Liu J., Liu M., Bai Y., Zhang J., Liu H., Zhu W. (2020). Recent Progress in Flexible Wearable Sensors for Vital Sign Monitoring. Sensors.

[20] Beck S., Laufer B., Krueger-Ziolek S., Moeller K. (2020). Measurement of Respiratory Rate with Inertial Measurement Units. Current Dir. Biomed. Eng..

[21] Xu D., Yu W., Deng C., He Z.S. (2022). Non-Contact Detection of Vital Signs Based on Improved Adaptive EEMD Algorithm (July 2022). Sensors.

[22] Karacocuk G., Höflinger F., Zhang R., Reindl L.M., Laufer B., Möller K., Röell M., Zdzieblik D. (2019). Inertial Sensor-Based Respiration Analysis. IEEE Trans. Instrum. Meas..

[23] Jayarathna T., Gargiulo G.D., Lui G.Y., Breen P.P. (2022). Electrodeless Heart and Respiratory Rate Estimation during Sleep Using a Single Fabric Band and Event-Based Edge Processing. Sensors.

[24] Vanegas E., Igual R., Plaza I. (2020). Sensing Systems for Respiration Monitoring: A Technical Systematic Review. Sensors.

[25] Roudjane M., Bellemare-Rousseau S., Khalil M., Gorgutsa S., Miled A., Messaddeq Y. (2018). A Portable Wireless Communication Platform Based on a Multi-Material Fiber Sensor for Real-Time Breath Detection. Sensors.

[26] Cesareo A., Biffi E., Cuesta-Frau D., D’Angelo M.G., Aliverti A. (2020). A Novel Acquisition Platform for Long-Term Breathing Frequency Monitoring Based on Inertial Measurement Units. Med. Biol. Eng. Comput..

[27] Rahmani M.H., Berkvens R., Weyn M. (2021). Chest-Worn Inertial Sensors: A Survey of Applications and Methods. Sensors.

[28] Monaco V., Giustinoni C., Ciapetti T., Maselli A., Stefanini C. (2022). Assessing Respiratory Activity by Using IMUs: Modeling and Validation. Sensors.

[29] Chu M., Nguyen T., Pandey V., Zhou Y., Pham H.N., Bar-Yoseph R., Radom-Aizik S., Jain R., Cooper D.M., Khine M. (2019). Respiration Rate and Volume Measurements Using Wearable Strain Sensors. NPJ Digit. Med..

[30] Laufer B., Krueger-Ziolek S., Docherty P.D., Hoeflinger F., Reindl L., Moeller K. An Alternative Way to Measure Respiration Induced Changes of Circumferences: A Pilot Study. Proceedings of the 2020 42nd Annual International Conference of the IEEE Engineering in Medicine & Biology Society (EMBC).

[31] Hexoskin Hexoskin Smart Shirts-Cardiac, Respiratory, Sleep & Activity Metrics. https://www.hexoskin.com/.

[32] Feito Y., Moriarty T.A., Mangine G., Monahan J. (2019). The Use of a Smart-Textile Garment during High-Intensity Functional Training: A Pilot Study. J. Sports Med. Phys. Fitness.

[33] Elliot C.A., Hamlin M.J., Lizamore C.A. (2019). Validity and Reliability of the Hexoskin Wearable Biometric Vest during Maximal Aerobic Power Testing in Elite Cyclists. J. Strength Cond. Res..

[34] Villar R., Beltrame T., Hughson R.L. (2015). Validation of the Hexoskin Wearable Vest during Lying, Sitting, Standing, and Walking Activities. Appl. Physiol. Nutr. Metab..

[35] Kogan D., Jain A., Kimbro S., Gutierrez G., Jain V. (2016). Respiratory Inductance Plethysmography Improved Diagnostic Sensitivity and Specificity of Obstructive Sleep Apnea. Respir. Care.

[36] Rozevika A., Katashev A., Okss A., Mantyla J., Coffeng R., Lhotska L., Sukupova L., Lacković I., Ibbott G.S. (2019). On the Monitoring of Breathing Volume, Using Textile Strain Gauges. Proceedings of the World Congress on Medical Physics and Biomedical Engineering 2018.

[37] Bhatt S.P., Balte P.P., Schwartz J.E., Cassano P.A., Couper D., Jacobs D.R.J., Kalhan R., O’Connor G.T., Yende S., Sanders J.L. (2019). Discriminative Accuracy of FEV1:FVC Thresholds for COPD-Related Hospitalization and Mortality. JAMA.

[38] Laufer B., Murray R., Docherty P.D., Krueger-Ziolek S., Hoeflinger F., Reindl L., Moeller K. (2020). A Minimal Set of Sensors in a Smart-Shirt to Obtain Respiratory Parameters. IFAC-Pap..

[39] Laufer B., Kretschmer J., Docherty P.D., Möller K., Höflinger F., Reindl L. (2017). Sensor Placement in a Smart Compression Shirt to Measure Spontaneous Breathing. Biomed. Tech..

[40] Wheatley I. (2018). Respiratory Rate 4: Breathing Rhythm and Chest Movement. Nurs. Times.

[41] Zoumot Z., LoMauro A., Aliverti A., Nelson C., Ward S., Jordan S., Polkey M.I., Shah P.L., Hopkinson N.S. (2015). Lung Volume Reduction in Emphysema Improves Chest Wall Asynchrony. Chest.

[42] Ricoy J., Rodríguez-Núñez N., Álvarez-Dobaño J.M., Toubes M.E., Riveiro V., Valdés L. (2019). Diaphragmatic Dysfunction. Pulmonology.

[43] Garcia-Pachon E. (2002). Paradoxical Movement of the Lateral Rib Margin (Hoover Sign) for Detecting Obstructive Airway Disease. Chest.

[44] de Groote A., Verbandt Y., Paiva M., Mathys P. (2000). Measurement of Thoracoabdominal Asynchrony: Importance of Sensor Sensitivity to Cross Section Deformations. J. Appl. Physiol..

[45] Jayasinghe U., Hwang F., Harwin W.S. (2022). Comparing Loose Clothing-Mounted Sensors with Body-Mounted Sensors in the Analysis of Walking. Sensors.

